# Functional redundancy as a stabilizing principle in bacterial communities under antibiotic perturbation: mechanisms, trade-offs, and emerging frameworks

**DOI:** 10.3389/fmed.2026.1834295

**Published:** 2026-05-28

**Authors:** Jingnan Ge

**Affiliations:** School of Health and Biochemical Sciences, Royal Melbourne Institute of Technology, Melbourne, VIC, Australia

**Keywords:** antibiotic resistance, functional redundancy, horizontal gene transfer, microbial community stability, YAS framework

## Abstract

The widespread use of antibiotics has severely disrupted the structure of microbial communities, but the responses of these communities vary in different environments. Interestingly, even when the species composition changes, some microbial communities can still maintain crucial functions, a phenomenon known as “decoupling of structure and function.” Among them, functional redundancy (FR) - the characteristic that multiple microorganisms perform the same ecological function - is the key mechanism for maintaining this stability. This review focuses on how functional redundancy may enhance microbial community resilience under antibiotic perturbation. We first start from the insurance hypothesis and the YAS (yield - acquisition - stress) framework to explain the ecological principles behind functional redundancy, and explain how microorganisms allocate resources and make trade-offs in different environments. We systematically analyze the multi-level defense strategies of microorganisms at five levels, including: ecological niche differentiation at the species level, horizontal transfer of resistance genes at the genetic level, cross-feeding reconstruction of metabolic networks, dormancy strategies at the temporal dimension (seed bank), and population regulation mediated by bacteriophages. Methodologically, we review metatranscriptomic approaches for distinguishing active signals from residual DNA, structural entropy algorithms for inferring FR, and AI-based tools for identifying latent resistance genes. Evidence from ecosystems such as the gut, respiratory tract, soil, and wastewater suggests the broad relevance of functional redundancy, although its stabilizing effect depends on antibiotic type, exposure duration, initial community composition, and ecological context. Finally, we explore the application prospects of this principle in the construction of synthetic communities and the optimization of fecal microbiota transplantation, and point out the evolutionary costs that may accompany maintaining functional redundancy, which is an important challenge that future research needs to address.

## 1 Introduction

### 1.1 Microecological dilemmas under antibiotic perturbation: the conflict between chemotherapy and ecological homeostasis

The discovery and clinical application of antibiotics are the cornerstone of modern medicine. However, with the widespread use of antibiotics, people have begun to realize the “incidental damage” caused by the symbiotic microbial communities (1). These exogenous chemical disturbances have enriched antibiotic resistance genes through strong selective pressure and disrupted the original community structure of microorganisms, ultimately leading to dysbiosis (2). Clinical and environmental studies have revealed a core issue: different microbial communities respond differently to antibiotics. Some communities disintegrate rapidly after exposure to antibiotics, with significant declines in metabolic functions; while other communities undergo huge changes in species composition, yet still maintain the stability of key ecological functions (such as producing short-chain fatty acids (SCFAs) or degrading pollutants). As Van Hul et al. (3) emphasized when redefining “healthy intestines,” species diversity cannot explain the stability of this system. This suggests a need to shift the assessment focus from taxonomic diversity to functional stability.”

### 1.2 The stability paradox and structure-function decoupling

Traditional ecological theory suggests that reduced species diversity can weaken ecosystem functions. The difference is that some researchers have observed that under the influence of antibiotics, the species composition of the microbial community has undergone significant changes, but the metabolic output has remained relatively stable. This proves that the assessment method of species classification is difficult to comprehensively reflect the changes in ecological functions. Allison (4) proposed the YAS theory to explain this phenomenon: under antibiotic pressure, microorganisms need to balance growth and reproduction with stress responses. Due to the limited total resources, increasing the investment in stress responses will inevitably lead to a decrease in growth rate and a reduction in unnecessary metabolic activities. This approach results in changes in the species structure of the community, but by regulating resource allocation, the stability of core metabolic functions can still be maintained.

### 1.3 Functional redundancy: a core insurance mechanism of ecosystems

Functional redundancy (FR) is a key concept linking structural change with functional stability. In simple terms, as explained by the insurance hypothesis in ecology: when a dominant species responsible for a core function is eliminated due to antibiotics, other functionally similar but less sensitive to antibiotics “backup” species will promptly fill the gap, thus preventing the entire system from collapsing (5). Jiang et al. (6) further revealed through the introduction of the concept of “structural entropy” that FR is a multi-level complex network feature. It is worth noting that the predictive indicators constructed based on FR can more accurately predict how communities respond to disturbances than traditional indicators that rely on species abundance. Moreover, Klümper et al. (7)’s research also shows that communities with a high level of FR tend to build more effective biological barriers to resist the invasion of foreign resistant genes. At the same time, however, the stabilizing role of FR should not be treated as universal, because its strength may vary across disturbance regimes, ecological contexts, and the methods used to infer function. Therefore, FR should be viewed as a conditional stabilizing mechanism rather than a universally protective property, because its effectiveness depends on whether functionally similar but differentially tolerant taxa are present and able to respond under a given disturbance regime.

### 1.4 Scope of this review

Although the significance of functional redundancy has been widely recognized, its definition, quantification, and stabilizing role under antibiotic interference are not always interpreted consistently across the literature. The specific mechanism by which it maintains community stability under antibiotic interference remains unclear. The formation mechanism of this phenomenon is rather complex. For instance, some bacteria can exchange resistance genes with each other through horizontal gene transfer, thereby forming a kind of “resistance redundancy” within the community, where multiple bacteria possess similar resistance functions (8). However, antibiotics can also cause some bacteria to lyse and die, which may disrupt the original redundant structure and make these functions difficult to maintain (9). These contrasting observations suggest that FR may support community stability in some cases, but may also be constrained, reorganized, or even undermined under others. Because several related concepts are often discussed alongside FR, this review also distinguishes FR from response diversity, metabolic plasticity, genetic redundancy, and structure–function decoupling, in order to clarify which processes constitute FR itself, which enable it, and which represent parallel forms of stabilization. Against this background, this review goes beyond a descriptive summary of existing concepts by proposing an integrated framework for understanding how FR contributes to the stability of bacterial communities under antibiotic stress. Specifically, we connect ecological theory with multi-level microbial mechanisms, including gene flow, metabolic trade-offs, dormancy, and virome-mediated regulation, to explain how FR is maintained, reorganized, or weakened under disturbance. We further argue that FR should be understood not as a static property of community composition, but as a dynamic and context-dependent stabilizing process. Based on this perspective, the review also considers how emerging analytical approaches may support the quantification of FR and inform the design of more effective microbial community intervention strategies.

## 2 Theoretical framework: from taxonomic diversity to functional stability

In microbial ecology research, taxonomic composition alone is often insufficient to predict how bacterial communities respond to antibiotic interference. As Van Hul et al. (3) noted in revisiting the concept of a “healthy gut,” community stability is not determined solely by species richness, but by the capacity to sustain key metabolic functions such as short-chain fatty acid production and bile acid transformation. This functional perspective provides the theoretical basis for focusing on FR as a core stabilizing principle. However, FR should not be treated merely as overlap among taxa performing similar roles; rather, it must be understood through an integrated framework that connects ecological insurance, resource-allocation trade-offs, and multi-level mechanisms of functional compensation under antibiotic stress.

To avoid conceptual overlap, this review uses these related concepts at different explanatory levels. Functional redundancy (FR) refers to a community-level property in which multiple taxa can contribute to the same ecological function. Response diversity describes differences in antibiotic sensitivity or recovery trajectories among functionally similar taxa; it therefore determines whether FR can be expressed after disturbance, but is not FR itself. Genetic redundancy refers to overlap or exchange of functional genes, including ARGs, that may support FR, whereas metabolic plasticity describes the ability of taxa or metabolic networks to switch pathways and maintain functional outputs without necessarily relying on taxon-level replacement. Structure–function decoupling is treated as an empirical outcome, referring to stable function despite taxonomic turnover, that may arise from FR, metabolic plasticity, or broader community reorganization.

Accordingly, this section outlines the theoretical basis for examining how bacterial communities maintain, reorganize, or lose function across different disturbance contexts, while also recognizing that the explanatory power of FR may vary across ecological settings and disturbance regimes.

### 2.1 Ecological foundations: the insurance hypothesis and the portfolio effect

The concept of FR originates from the “insurance hypothesis” in ecology. This hypothesis explains that a higher level of biodiversity can ensure that the basic functions of the ecosystem are less likely to be disrupted during environmental changes. In bacterial communities subjected to antibiotic interference, if the dominant bacterial strain (such as those involved in cellulose decomposition) that originally performed a certain key function decreases due to its sensitivity to antibiotics, other bacteria with similar functions but greater tolerance to antibiotics may increase. These bacteria can perform similar metabolic functions, and the overall function can still remain relatively stable (10).

This process is similar to the “portfolio effect” in finance. If an ecosystem overly relies on a highly efficient species, and that species becomes particularly sensitive to a certain antibiotic, the system will face a higher risk. On the contrary, communities with higher FR maintain stability by “diversifying risks,” that is, they contain multiple bacterial populations with similar functions but different compositions of resistance genes, thereby reducing the possibility of the entire system’s function collapse (11).

Cornell et al. (12) found in their study of the impact of land use changes on microbial communities that although environmental disturbances might reduce the number of species in certain areas, the coexistence networks formed among the surviving species might become more complex and tightly connected. This reorganization of the network structure can be regarded as an adaptive adjustment of the microbial community to environmental pressures (including antibiotic pressures). Therefore, even if antibiotic interference leads to changes in the community classification structure, as long as key functions can continue to be maintained through different species and their relationships, the overall function may still remain relatively stable. This situation is often referred to as “classification-function decoupling.” However, structure–function decoupling should not automatically be equated with FR, because similar functional outcomes may also arise from metabolic plasticity, shifting interactions, or other forms of community reorganization. Likewise, response diversity and genetic redundancy may support FR, but they are not interchangeable with FR itself.

### 2.2 The YAS framework: resource allocation trade-offs under antibiotic stress

This redundancy does come at a cost. Allison (4)’s YAS theory explains this limitation. According to this theory, microorganisms need to make trade-offs among three aspects when using resources: first, growth efficiency, which refers to the ability to synthesize biomass; second, resource acquisition, such as secreting extracellular enzymes to break down and utilize external substances; and third, stress resistance, such as repairing damage or eliminating toxic substances. Due to limited resources, none of these three abilities can reach their peak simultaneously. In the absence of antibiotic pressure, the community is usually dominated by microorganisms with higher growth efficiency (13); under antibiotic intervention, the survival strategy of the community must be adjusted accordingly.

**FIGURE 1:**
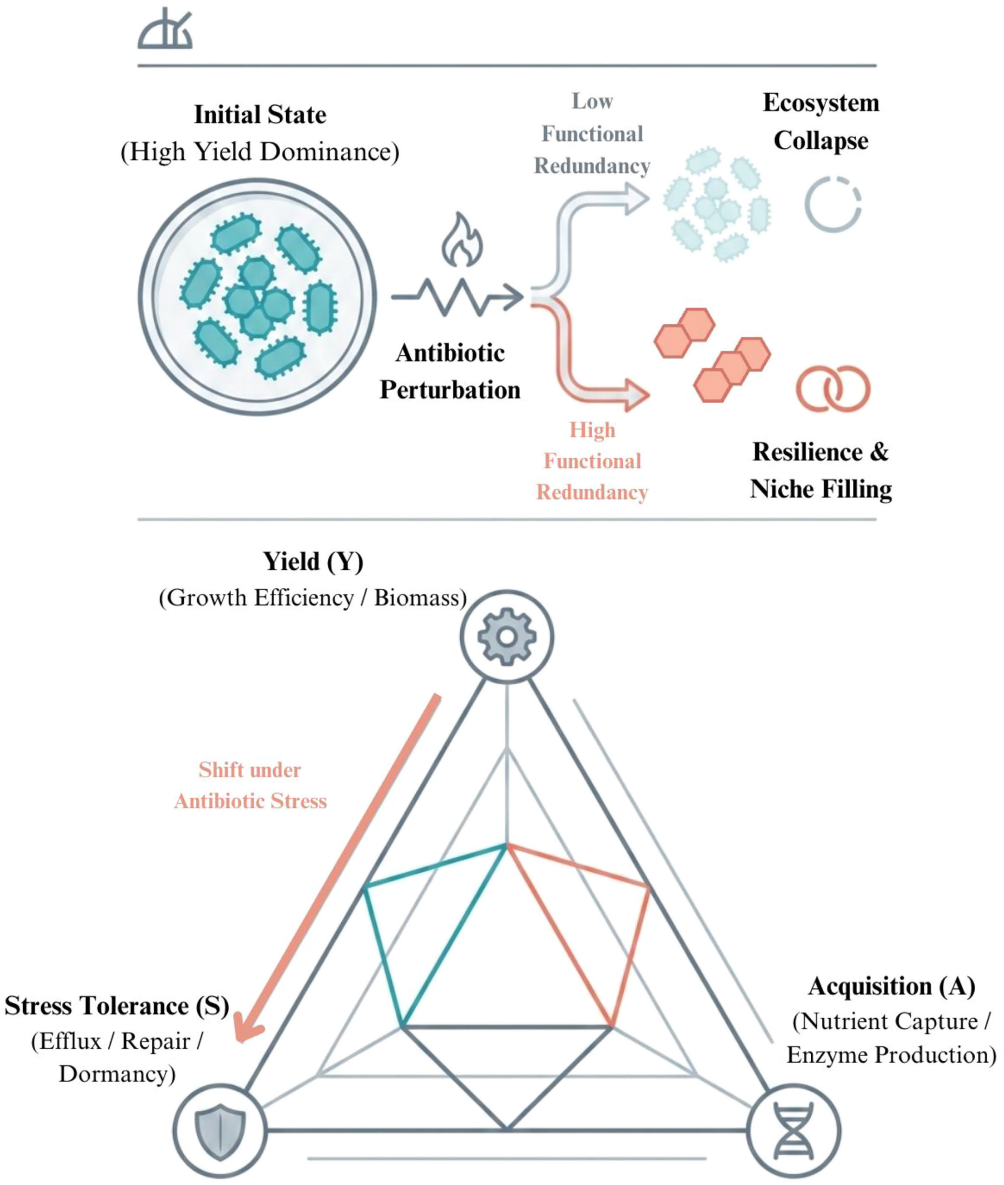
Conceptual framework of FR-mediated stability and YAS trade-offs.

To illustrate this ecological mechanism and its influence on community trajectory, we constructed a conceptual model in Figure 1. The upper panel of Figure 1 shows that communities with different levels of FR may follow contrasting successional outcomes after antibiotic disturbance. On the left, low-redundancy communities experience ecosystem collapse due to the absence of key functional groups and their lack of replenishment. On the right, high-redundancy communities experience the disappearance of dominant groups but are quickly filled by resistant redundant groups, thereby maintaining functional recovery.

More importantly, the lower panel of Figure 1 illustrates the mechanism underlying this divergence by showing how survival strategies are reallocated within the YAS framework. Under antibiotic pressure, bacteria need to shift limited energy from “output” (Yield) in the YAS triangle to stress tolerance, such as enhancing the expression of efflux pumps, synthesizing components of biofilms, or entering a dormant state. This resource redistribution indicates an important cost behind FR: bacteria with similar functions but greater tolerance are usually less efficient, thus leading to a decline in the overall metabolic level over time. This also explains a situation often observed in research: after antibiotic treatment, the microbial community can still perform certain functions, such as inhibiting the colonization of pathogens. However, compared to the situation without treatment, their efficiency in nutrient utilization and metabolism is usually reduced. Therefore, the stability brought about by FR is essentially an ecological trade-off, that is, by reducing short-term growth and metabolic efficiency in exchange for the long-term survival of the community. In this sense, the YAS framework provides the ecological logic for understanding why FR can be maintained under antibiotic stress, but also why such stability is often partial and conditional. The multi-level mechanisms discussed in the following section can be understood as the specific pathways through which these trade-offs are translated into functional compensation, reorganization, or failure. At the same time, the extent to which such compensation is realized likely depends on disturbance intensity, community composition, and the methods used to infer functional persistence.

### 2.3 Disturbance regimes: pulse versus press antibiotic stress

The extent to which FR can exert its effects largely depends on the manner in which antibiotics interfere. If antibiotics are used in a “pulsed interference” manner, that is, in short periods of high doses, such as in a one-time clinical treatment, the ability of the community to recover mainly depends on its recovery capacity. FR is like a reserve force: some originally weak microorganisms will rapidly resume growth, helping the community recover and reducing the chance of pathogen invasion. On the contrary, if antibiotics are used in a “stressful interference” manner, that is, in a long-term, low-dose presence, such as continuous exposure in a livestock environment. FR usually operates through horizontal gene transfer (HGT), that is, microorganisms with similar metabolic functions exchange resistance genes, enabling the entire population to enhance its long-term tolerance to antibiotics. Klümper et al. (7) pointed out that under this long-term interference, if the community has a high degree of FR, the competition between different species will be stronger. This competition can, to a certain extent, disperse the impact of antibiotics, helping to maintain the functional stability and diversity of the community. Taken together, these patterns suggest that FR is most effective under disturbance regimes in which functionally similar but differentially tolerant taxa remain available for compensation and where recovery pathways are not interrupted. By contrast, its stabilizing effect may be weakened under chronic low-dose selection, strong metabolic trade-offs, or top-down disruptions such as phage-mediated collapse. More broadly, the contribution of FR to community stability is expected to vary with antibiotic class, exposure duration, initial community composition, availability of functionally similar taxa, and the ecological context in which disturbance occurs.

## 3 Core mechanisms underlying FR-mediated stability

**FIGURE 2:**
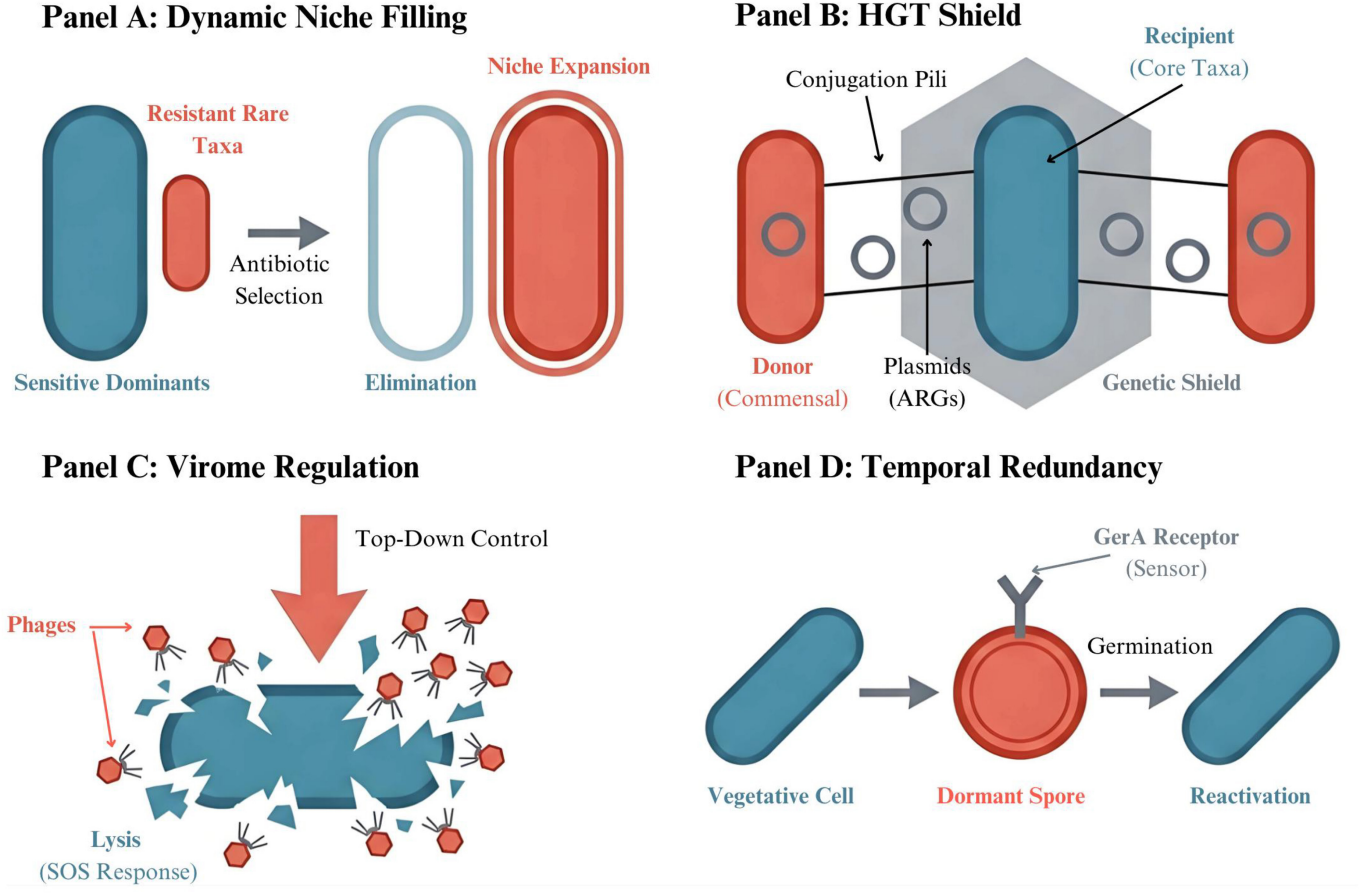
Multidimensional mechanisms underpinning FR-mediated stability. **(A)** Dynamic Niche Filling: Under antibiotic selection, sensitive dominant taxa are eliminated, while functionally similar resistant taxa (rare biosphere) expand to occupy the vacant niche, compensating for functional loss. **(B)** HGT-Mediated Shielding: Commensal bacteria act as reservoirs for resistance genes, transferring plasmids to core metabolic taxa via conjugation, thereby forming a “genetic shield” against antibiotics. **(C)** Virome Top-Down Regulation: Antibiotic stress triggers the SOS response and prophage induction, leading to host lysis and imposing top-down control that can destabilize the community. **(D)** Temporal Redundancy: Bacteria escape lethal stress by entering dormancy (sporulation) and form a seed bank. Reactivation is triggered by environmental sensing via specific receptors (e.g., GerA) once conditions improve.

Functional redundancy is a multi-dimensional dynamic defense mechanism. Mechanistically, FR should not be understood as a single process, but as an emergent property of how bacterial communities respond to antibiotic stress through multiple compensatory pathways. In this context, the YAS framework provides the ecological logic for why resource reallocation occurs under disturbance, whereas the mechanisms discussed below represent the specific pathways through which FR is maintained, reorganized, or disrupted. Under antibiotic intervention, bacterial communities may maintain or partially recover functional stability through various mechanisms, including ecological niche substitution among different species, horizontal gene transfer at the genetic level, reorganization of metabolic networks, and dormancy-based strategies. However, the extent to which these mechanisms support FR depends on antibiotic pressure, community composition, metabolic costs, and ecological interactions. These processes unfold along the temporal dimension. Meanwhile, this network may also be constrained by viral top-down regulation. Importantly, however, these mechanisms should not be assumed to operate with equal strength across all communities, because their effectiveness depends on ecological context, disturbance regime, and the composition of the resident microbiota. To visually illustrate these interactions, we have drawn Figure 2, in which Panels A–D summarize the main compensatory and disruptive pathways discussed below as a multi-level defense model.

### 3.1 Response diversity and changing niche filling

For FR to be effective, there is an important prerequisite: there must be certain differences in responses within the group. At the community level, this means that YAS-mediated shifts in survival strategy do not affect all functionally similar taxa equally, thereby allowing more tolerant populations to compensate for sensitive ones. That is to say, the sensitivity of microorganisms with similar functions to antibiotics is not the same. The traditional view holds that when the main bacteria that are sensitive to antibiotics are eliminated, those bacteria with similar functions but resistance will increase rapidly due to reduced competition. Lyng and Kovács (14) proposed the concept of “frenemies,” indicating that this process is actually much more complex because the relationships between microorganisms change with environmental conditions.

Without pressure, functionally similar bacteria usually compete for resources. For example, in soil, *Bacillus* and *Pseudomonas* often compete for the same nutrients. But when antibiotics are present, they sometimes turn to cooperation. Some resistant bacteria may weaken the effect of antibiotics by secreting polysaccharides or producing degrading enzymes, thereby protecting the more sensitive bacteria to antibiotics to a certain extent. As illustrated in Figure 2A, this process allows resistant but functionally similar taxa to occupy the vacant niche left by sensitive dominant groups, thereby supporting functional compensation. Nevertheless, such compensation is unlikely to be universal, because niche filling depends on whether functionally similar but differentially tolerant taxa are actually present and able to expand under the given ecological conditions.

### 3.2 Mobile resistomes: HGT–mediated resistance redundancy

Functional redundancy is also manifested in the rapid spread of antibiotic resistance genes (ARGs) within microbial communities. At the genetic level, one important way to resolve the trade-offs imposed by antibiotic stress is through HGT, which allows key functions to persist even when the original taxa are vulnerable. O’Connor and Heyderman (8) discovered while studying the nasopharyngeal microbiome that many symbiotic bacteria are actually important repositories of mobile genetic elements (MGEs). Under antibiotic pressure, some bacteria responsible for core metabolic functions can transfer through conjugation and acquire plasmids carrying resistance genes from these symbiotic bacteria.

This process belongs to HGT, which enables different species to protect their originally vulnerable metabolic networks by sharing resistance mechanisms. Figure 2B illustrates this mechanism as a “genetic shield,” in which MGEs move through the community network and help preserve the survival of key functional taxa. This also explains why some core ecological functions still maintain a strong recovery ability under long-term antibiotic exposure. At the same time, this mechanism also highlights an important tension in the literature: although HGT may stabilize community function, it may also promote resistome expansion, meaning that functional persistence is not always ecologically or clinically beneficial.

### 3.3 Metabolic plasticity and cross-feeding

The third important role of FR is to maintain the stability of microbial metabolic functions. At the metabolic-network level, FR can be sustained when antibiotic-induced resource reallocation promotes alternative pathway use and cross-feeding interactions among taxa. Bacterial communities usually have strong metabolic flexibility and can complete similar metabolic processes in different ways. When antibiotics block certain major metabolic pathways or inhibit key bacterial species, other microorganisms in the community can continue to maintain the overall function by initiating alternative metabolic pathways or strengthening nutrient exchange between different species (15).

If the functions of primary decomposing bacteria are impaired, other fermentation bacteria will activate alternative metabolic pathways and utilize the remaining degradable substances in the environment to continue synthesizing SCFAs. Although not shown as a separate panel, this metabolic reorganization is conceptually embedded in Figure 2 as part of the network-level compensation that links taxonomic turnover to functional persistence. This observation suggests that community stability is closely related to microbial complementarity. However, evidence for such compensation is often inferred from community-level patterns rather than directly demonstrated, and the extent to which cross-feeding fully restores function may differ across systems.

### 3.4 Temporal redundancy: dormancy and seed banks

Functional redundancy is also manifested in the temporal dimension. At the temporal level, FR may be preserved through dormancy, which acts as a delayed compensation mechanism when active community members cannot immediately withstand antibiotic stress. Moir (16)’s molecular research provides evidence for this. It shows that when the antibiotic concentration is very high, some bacteria temporarily cease their functional activities in the community by forming spores or entering an almost inactive “hibernation state” (Figure 2D). These bacteria in the hibernation state are like a “seed bank,” preserved in the community.

Moir also found that some germination receptors (such as the GerA receptor) can sense signals indicating weakened antibiotic pressure or restored nutritional conditions, and then trigger these dormant bacteria to start growing again. As illustrated in Figure 2D, dormancy therefore functions as a temporal reservoir of compensatory potential, allowing FR to be preserved even when active taxa are temporarily lost. Thus, even if most active bacteria are eliminated, the community still retains a part of “backup power,” which can quickly restore its original function when the environment improves. Even so, dormancy should be interpreted as preserving recovery potential rather than guaranteeing recovery itself, because successful reactivation depends on subsequent environmental conditions and community reassembly dynamics.

### 3.5 Virome-mediated top-down regulation: the overlooked disruptor

Although these mechanisms may stabilize community function, their compensatory effects are not unlimited, and one potentially important but still under-characterized constraint is phage-mediated top-down control. The virome may act as a force that disrupts FR. Tun et al. (9) discovered in their study of enteric viruses that antibiotics induce the SOS response in bacteria, thereby triggering the induction of bacteriophages and entering the lysis cycle.

This lysis mediated by bacteriophages leads to the synchronous destruction of the dominant symbiotic community as shown in Figure 2C, triggering a chain of community collapse. Therefore, the top-down regulation driven by bacteriophages constitutes a negative control over FR. If this influence is not taken into account, then when evaluating the impact of antibiotics on microbial stability, the community’s recovery ability may be seriously overestimated. Current evidence suggests that phage-mediated disruption can be ecologically significant, especially in host-associated microbiomes, but its magnitude, generality, and consequences for FR across ecosystems remain insufficiently resolved. Figure 2C highlights this pathway as a direct interruption of functional compensation, showing how FR may become vulnerable when virome-driven mortality removes functionally compensatory taxa. This suggests that FR may be particularly vulnerable in systems where prophage induction, host susceptibility, or virome-driven mortality disrupt the persistence of functionally compensatory taxa.

## 4 Quantification strategies and methodological advances

Assessing FR is important for understanding microbial community stability. With advances in sequencing and computational biology, methods for evaluating microbial functions have improved substantially. Early studies mainly measured functional gene abundance, whereas newer approaches, including microbial network analysis and AI-based prediction, provide additional ways to infer which microbial groups may contribute to key functions under disturbance. These technologies are useful in antibiotic-related studies because they help distinguish potentially present genes from actively expressed signals, while reducing bias from residual DNA.

However, these approaches represent different levels of functional inference rather than direct measurements of realized ecological function. Metagenomics mainly reflects potential functional capacity, metatranscriptomics provides a proxy for expressed activity, structural entropy captures the inferred organization of FR, and AI-based prediction expands candidate annotations. None of these methods can replace direct validation through metabolite measurements, process-rate assays, cultivation-based experiments, or controlled perturbation tests.

### 4.1 From metagenomics to metatranscriptomics: from potential redundancy to expressed activity

When studying antibiotic effects on microbial communities, relying only on DNA metagenomic sequencing can be misleading. Antibiotics may kill or damage many bacteria, but their DNA can persist in the environment. As a result, DNA sequencing may detect genetic information from both living and dead cells, making the community appear to have high FR and recovery potential even when some functions are no longer active (17). Thus, DNA-based inference may overestimate FR when detected genes represent residual potential rather than ongoing ecological activity. Metagenomic evidence should therefore be interpreted as potential redundancy, not realized functional redundancy, because gene presence does not necessarily indicate gene expression or maintained ecological processes.

For this reason, some studies combine metagenomics with metatranscriptomics. Because RNA generally reflects genes that cells are actively using, RNA transcripts can help identify which functions may remain active under antibiotic pressure. Joint DNA and RNA analysis therefore helps distinguish potential functional capacity from expressed functional potential. Even so, RNA abundance should still be interpreted cautiously, because transcriptional activity is better viewed as a proxy for expressed functional potential rather than realized ecological function, especially under antibiotic stress or dormancy. Direct evidence of realized function still requires complementary measurements such as metabolite profiles, enzyme activity, nutrient transformation rates, or experimentally validated community outputs.

### 4.2 Network topology and structural entropy: complementing abundance-based metrics

Early methods for evaluating FR mainly relied on species number or functional gene copy number, but they ignored interactions among taxa. Traditional metrics such as species richness, diversity indices, and functional gene abundance are useful for estimating potential functional capacity, but they do not capture how taxa are connected or how functional compensation is organized. Ecological network analysis therefore provides a complementary perspective. For instance, Cornell et al. (12) found that under disturbance, such as antibiotics or land-use change, microbial taxa may decrease, while associations among remaining taxa may become closer, partly compensating for diversity loss.

Based on these observations, Jiang et al. (6) proposed Structural Entropy as a network-based indicator. Unlike common entropy indices, Structural Entropy treats the microbial community as a hierarchical network and describes how functionally related taxa are organized and connected. The research team also identified functional redundancy clusters (FRC), in which different species show functional complementarity and close interconnections (18). Compared with abundance-based metrics, FR estimated by Structural Entropy may detect community reorganization earlier and may better reflect the inferred organization and stability of functionally redundant groups. However, these advantages should be interpreted cautiously, because structural entropy depends strongly on network construction, edge and cluster definitions, and multi-omics data quality. It should therefore be viewed as complementary to, rather than a replacement for, traditional abundance-based and diversity-based metrics. Moreover, structural entropy can help infer how FR is organized, but it does not by itself demonstrate that redundant functions are realized under antibiotic stress. Its interpretation should be combined with expression-level, metabolite-level, or process-level evidence whenever possible.

**FIGURE 3:**
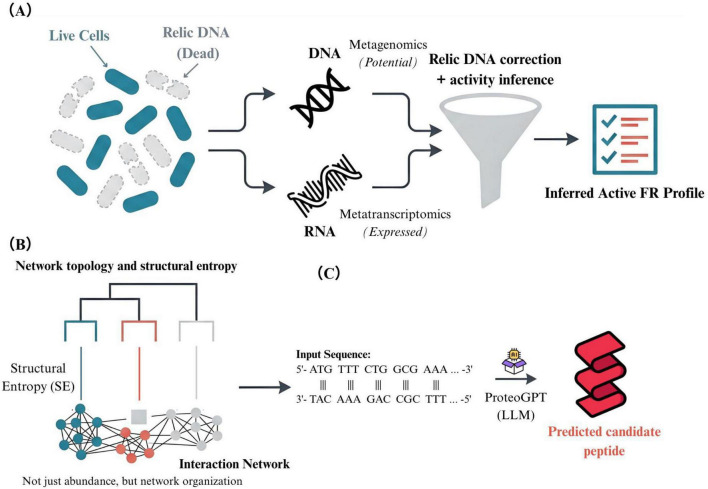
Advanced workflow for inferring and predicting FR: from abundance-based inference and structural entropy analysis to AI-based prediction. **(A)** Parallel analysis of metagenomic DNA and metatranscriptomic RNA, combined with relic DNA correction, is used to infer active FR while reducing bias from dead-cell DNA. **(B)** Structural entropy is applied to hierarchical functional networks to capture the organization and stability of functionally redundant groups beyond abundance-based metrics. **(C)** AI-based prediction expands candidate functional discovery, although these predictions require cautious interpretation and experimental validation.

To illustrate this methodological progression, Figure 3 summarizes how Panel A distinguishes potential and expressed activity, Panel B captures the inferred network organization of FR beyond abundance-based metrics, and Panel C expands candidate functional discovery through AI-based prediction.

Figure 3 illustrates a workflow for inferring and predicting FR through multidimensional datasets: (1) Step 1: Parallel analysis of metagenomic DNA and metatranscriptomic RNA. In antibiotic-related studies, RNA/DNA correction can reduce relic DNA-derived artifacts and generate an inferred profile of expressed FR. (2) Step 2: Based on Jiang et al. (6), structural entropy algorithms construct hierarchical functional networks. Taxa are classified into the same FRC according to functional similarity, and cluster stability serves as an indicator for inferring recovery potential. (3) Step 3: Based on Wang et al. (19), generative AI models such as ProteoGPT can predict candidate functional genes from unidentified metagenomic sequences, including new resistance determinants or antimicrobial peptides, thereby expanding the predicted range of FR. Nevertheless, this workflow should be viewed as a promising analytical framework rather than a standardized solution, because each step introduces uncertainty and cannot replace direct validation of realized ecological functions.

### 4.3 AI-based functional discovery: large language models as a new tool

The traditional FR quantification methods are constrained by existing gene databases, which merely contain well-studied resistance mechanisms, such as common β-lactamases or efflux pumps. When new genetic changes occur in bacterial communities under antibiotic pressure, such as the emergence of previously unidentified resistance genes or new stress-resistant proteins, these components are easily ignored in the analysis. This situation is common in environmental microbial communities because massive genes have unclear functions. To solve this problem, new methods are necessary to explore these functions (20).

Wang et al. (19) developed ProteoGPT, which utilizes LLMs to identify functional genes. This approach regards protein sequences as a “biological language” and infers their functions by learning patterns and structural information from many sequences. It means that even if a gene is not highly similar to known sequences, as long as its structural features conform to certain typical functional patterns, the gene can still be identified (21), such as the catalytic structure of a resistance enzyme or the amphipathic helix structure of an antimicrobial peptide. This method is particularly suitable for discovering candidate stress response genes activated under antibiotic pressure, which differ from the known genes in the database. At the same time, AI-based functional prediction should not be interpreted as direct functional confirmation, because inferred annotations still require careful experimental validation. Therefore, AI-based prediction should be understood as a hypothesis-generating approach for identifying candidate redundant functions, rather than as direct evidence that these functions are realized in the community.

In the process, Wang and his team discovered previously unknown antibacterial peptides in natural microbial communities and demonstrated how to design new functional proteins (19). When studying genomic changes under antibiotic stress, if this LLM-based analytical method is combined with traditional methods, there is a chance to discover ignored redundant genes, thereby improving the assessment of genetic potential and environmental adaptability of bacterial communities. More broadly, the value of these tools lies in hypothesis generation and expansion of the searchable functional space, but their outputs may still be shaped by training-data bias, annotation incompleteness, and uncertainty in biological interpretation.

## 5 Empirical evidence across biomes

As a central mechanism underpinning community stability, FR exhibits pronounced context dependency across ecosystems. From the high-density gut microbiome to low-biomass respiratory habitats, and further to complex soil and aquatic environments, responses of FR to antibiotic pressure share common principles, yet differ markedly in their dominant implementation pathways, whether through species replacement, horizontal gene transfer, or metabolic trade-offs. At the same time, the available evidence does not support a single universal mode of FR-mediated stability, because both the dominant mechanisms and the resilience outcomes vary across biomes and disturbance contexts. In some systems, FR appears to facilitate functional compensation, whereas in others it may be weakened, reorganized, or overridden by top-down disruption and longer-term trade-offs. Notably, representative evidence across biomes is summarized in Table 1, with an additional indication of evidence type to distinguish direct experimental evidence from observational, theoretical, or review-based support. These examples therefore support FR as a useful but context-dependent framework, rather than as a universal predictor of resilience across all antibiotic-perturbed communities.

**TABLE 1:** Summary of empirical studies examining FR responses to antibiotic pressure across biomes.

Reference	Evidence type/strength	Biome	Stressor/ context	Key mechanism	Impact on FR	Resilience outcome
Allison (4)	Conceptual/ theoretical framework	Soil	Drought and antibiotics	YAS trade-off: resource allocation shifts from growth to stress tolerance (EPS production, dormancy)	Maintained but suppressed: FR preserves survival, but functional rates (yield) decline	High resistance: community persists, carbon cycling slows
O’Connor and Heyderman (8)	Review-based evidence	Nasopharynx	Antibiotic treatment	HGT/MGEs: commensals act as ARG reservoirs via mobile elements	Genetic redundancy: FR maintained through gene sharing rather than species replacement	Structural stability: biofilms persist, resistome expands
Tun et al. (9)	Review-based/ observational evidence	Gut	Antibiotics (dysbiosis)	Prophage induction: SOS-triggered entry into the lytic cycle	FR collapse: redundant taxa lost through viral lysis (“domino effect”)	Low resilience: dysbiosis persists, colonization resistance fails
Klümper et al. (7)	Experimental/ environmental microbiome evidence	Environment/ Soil	ARG invasion	Biotic resistance: diversity-mediated competition	Barrier effect: high FR prevents invasion of new ARGs/plasmids	High stability: invasion blocked, native community protected
Lyng and Kovács (14)	Review-based/ model-system evidence	Soil models	Co-culture interactions	Response diversity: “frenemies” dynamics (competition ↔ cooperation)	Niche filling: dynamic interactions enable rapid niche reoccupation	Functional recovery: metabolic networks re-established

The evidence type column highlights that current support for FR-mediated stability comes from different levels of evidence, including conceptual frameworks, review-based synthesis, observational findings, and experimental environmental microbiome studies. Therefore, the table should be interpreted as a comparative summary of representative evidence rather than as a direct ranking of causal strength across ecosystems.

### 5.1 Host-associated microbiomes: colonization resistance and resistome reservoirs

#### 5.1.1 The gut ecosystem

In an environment with extremely high microbial density such as the intestinal tract, FR often manifests as a form of “colonization resistance.” In simple terms, a healthy gut microbiota will occupy the space within the intestine and consume most of the available nutrients, thereby preventing the colonization of foreign harmful bacteria. If antibiotics reduce some of the originally dominant symbiotic bacteria, such as the *Bacteroides* genus, then communities with strong colonization resistance can often make up for it by increasing the number of other bacteria. For example, some secondary fermentation bacteria (such as the *Clostridium* cluster) may become more abundant, thereby continuing to maintain the production of SCFAs and also being able to prevent opportunistic pathogens (such as *Clostridium difficile*) from entering and multiplying in large numbers.

However, this recovery ability does not always remain constant. Tun et al. (9) pointed out that the viral community in the gut also has a significant impact on this process. Antibiotics may trigger the activation of a large number of bacteriophages, which will infect and destroy bacteria. If this situation is severe, it may consume the bacteria that could have played a supplementary role, resulting in a significant decline in colonization resistance and even complete failure. This suggests that bacteriophages may act as a top-down constraint on FR-mediated stability in the gut. However, direct evidence linking phage induction to redundancy loss remains limited.

#### 5.1.2 Respiratory and nasopharyngeal ecosystems

Compared to the intestines, the microbial population in the nasopharynx is much smaller, and thus is regarded as a “low biomass” ecosystem. In such an environment, FR often relies on genetic-level changes. O’Connor and Heyderman (8) discovered that some commensal bacteria in the nasopharynx, such as *Dolosigranulum* and *Moraxella*, actually carry a large number of antibiotic resistance genes (ARGs) and MGEs. When antibiotic pressure occurs in the environment, these non-pathogenic bacteria sometimes transfer resistance genes through HGT to other bacteria in the community, including some core metabolic bacteria, and even to pathogens like *Streptococcus pneumoniae* (22).

Although this kind of gene sharing makes pathogens more likely to acquire drug resistance, it can also help the microbial biofilms in the nasopharynx maintain their overall structure under the influence of antibiotics. O’Connor and Heyderman (8) pointed out that this “genetic redundancy” driven by MGEs enables such microbial communities, which already have a relatively small number of members, to remain basically stable when subjected to antibiotic interference, while still continuing to participate in regulating the host’s immune response.

### 5.2 Environmental microbiomes: stress tolerance and biotic barriers

#### 5.2.1 Soil ecosystems and drought analogies

In the soil environment, microbial communities often exhibit similar responses to various environmental stresses such as antibiotics, drought, or nutrient deficiency. Therefore, if a microbial community has adapted to one type of stress, it is usually more likely to cope with other stresses. This is important for understanding FR in the context of antibiotics. Allison (4) compared the responses of bacteria to antibiotics and drought. He found that when deprived of water or exposed to antibiotics, bacteria typically secrete extracellular polymers (EPS) to form a protective layer, while reducing their growth rate and entering a “energy-saving” state.

Under the influence of environmental pressures such as antibiotics, only a small number of microorganisms with stronger tolerance capabilities (such as actinomycetes) can continue to survive. However, in order to maintain basic survival, these microorganisms usually reduce the metabolic activities related to carbon and nitrogen element transformation. Therefore, although the soil system will not collapse, certain ecological functions will still decline in the short term.

This phenomenon has also been verified in experiments. Lyng and Kovács (14) found after culturing various soil bacteria together that when antibiotics were present in the culture medium, some bacteria that had previously competed with each other (such as *Bacillus* and *Pseudomonas*) would adjust their original interaction and jointly produce protective substances. This change enhanced the overall community’s ability to resist interference.

#### 5.2.2 Wastewater treatment plants

As a hotspot area for the accumulation of antibiotic residues, the activated sludge system in wastewater treatment plants becomes a laboratory for testing the FR theory. Klümper et al. (7) research indicates that the high species diversity in the activated sludge forms a powerful biological barrier, preventing the invasion and colonization of alien multi-drug resistant plasmids. The high FR ensures the continuous removal of organic pollutants and nutrients (nitrogen and phosphorus), and also dilutes the selection pressure of antibiotics through intense interspecies competition, thereby stopping the outbreak of a single dominant drug-resistant clone (23).

## 6 Challenges and future perspectives

Taken together, the evidence reviewed here suggests that FR should be understood not as a static property of community composition, but as a dynamic and context-dependent stabilizing process under antibiotic perturbation. By integrating ecological theory, resource-allocation trade-offs, and multi-level microbial mechanisms, this review highlights that the capacity of FR to buffer disturbance depends not only on the presence of functionally similar taxa, but also on the costs, constraints, and boundary conditions that shape their persistence and compensation. Although these stabilizing processes may be crucial for maintaining microbial community function, their application in ecological engineering and clinical intervention still faces two major challenges: the evolutionary cost of redundancy and the current limitations of quantification and design tools. Future research should therefore move beyond simply identifying FR, and instead focus on how to define, measure, optimize, and maintain FR under different disturbance regimes.

### 6.1 The cost of redundancy: evolutionary filtering under stress-free conditions

Functional redundancy is not always beneficial. Maintaining this redundancy requires microorganisms to continuously incur costs, and the cost level depends on the environmental conditions. According to the YAS framework proposed by Allison (4), a community with multiple stress-resistant mechanisms and FR must continuously invest resources to maintain these resistance structures - such as continuously expressing efflux pumps or synthesizing biofilm matrix - even if there is no antibiotic threat around, this investment cannot be stopped. This continuous metabolic consumption makes redundant communities difficult to compete with those that focus on growth and reproduction and have efficient metabolism in stable and undisturbed environments.

This trade-off influences the rational use of antibiotics in clinical treatment and in agricultural production. Taking the hospital environment as an example, if there is long-term exposure to low-dose antibiotics, the microbial community might develop higher FR with multiple strains with resistance (24). Although these communities are functionally stable, they may also become long-term storage repositories for antibiotic-resistant genes because the selection pressure always exists and resistance will not be eliminated. In contrast, if the environment is restored after the use of antibiotics, the microorganisms tend to abandon those redundant functions that consume resources, but this simplification may also lead to a decrease in the community’s resistance to subsequent disturbances.

The future microbial group engineering needs to address the issue of balancing the contradiction between stability and efficiency. That is to say, the artificially designed or transplanted microbial communities should not only retain sufficient FR to cope with sudden antibiotic impacts, but also avoid losing normal ecological functions during the recovery period due to excessive burden. This trade-off further supports the view that FR is not universally beneficial, but operates within ecological and evolutionary limits that must be explicitly considered in both theory and application.

### 6.2 Synthetic ecology and microbiome engineering: a decentralized design paradigm

**FIGURE 4:**
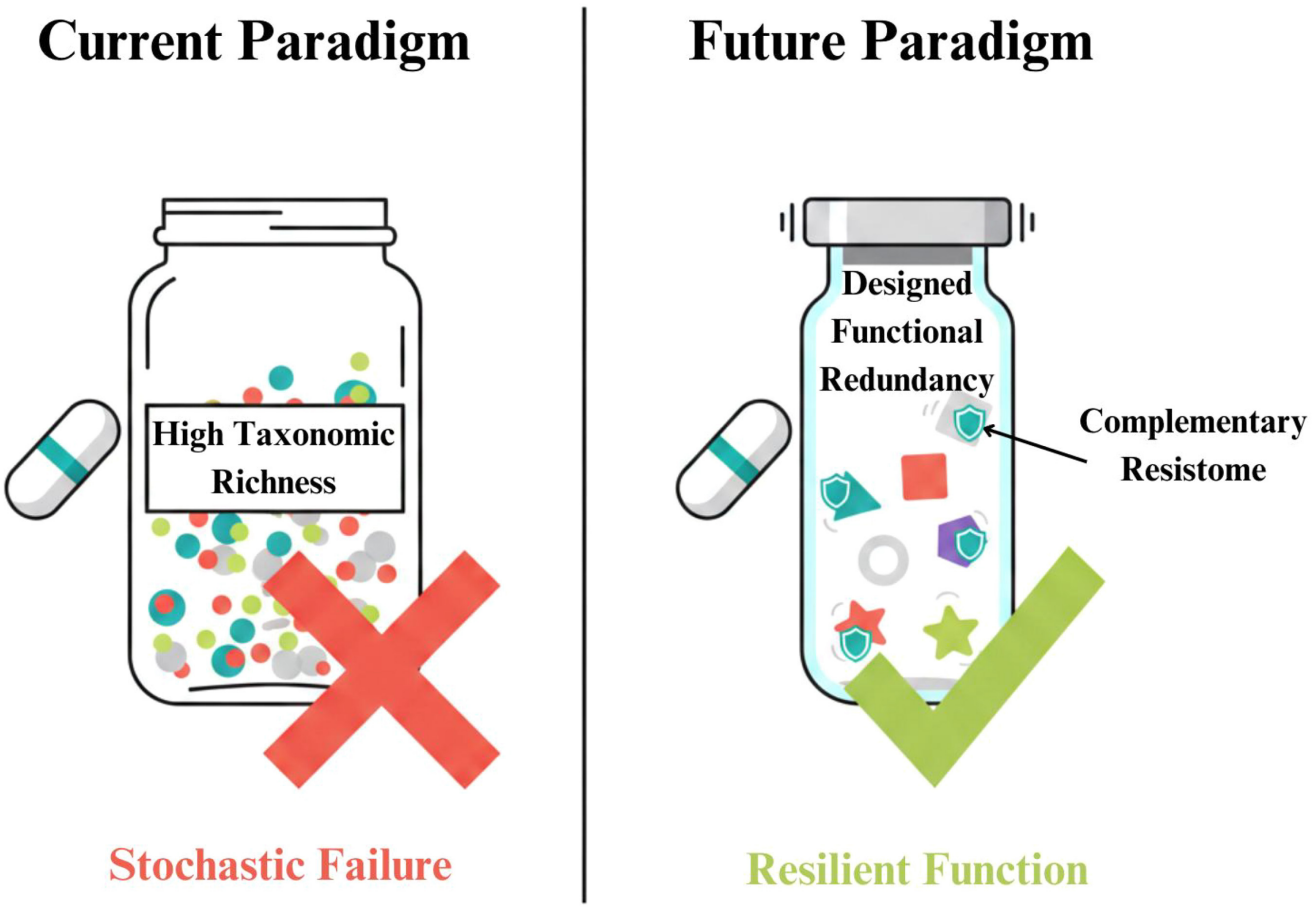
Comparative paradigms for microbiome intervention strategies.

Traditional microbial population engineering often aims to identify a “super strain” that can independently perform a specific function, such as pollutant degradation or probiotic activity. However, under antibiotic pressure, a single strain is often vulnerable to disturbance and may fail to provide sustained functional output. From the perspective developed in this review, a more robust strategy is to design microbial communities around FR rather than taxonomic dominance. Klümper et al. (7) suggested that this can be achieved through synthetic microbial communities, in which multiple strains jointly support the same key ecological function while differing in stress tolerance and survival strategies. Figure 4 illustrates this shift by contrasting the current richness-centered paradigm with a future function-centered and redundancy-informed design strategy.

In the current approach (left side of Figure 4), interventions often rely on complex microbial communities that function largely as a “black box.” Traditional fecal microbiota transplantation (FMT) or multi-strain probiotic products, for example, may contain many microorganisms, but they are usually not specifically designed to withstand antibiotic stress (25–27). Once broad-spectrum antibiotics are used, key taxa may be lost, leading to unstable treatment effects. As illustrated on the left side of Figure 4, high taxonomic richness alone does not necessarily ensure stability and may still lead to stochastic failure.

In contrast, the synthetic ecology approach (right side of Figure 4) emphasizes function-based design. Researchers can use artificial intelligence to select a small number of strains that perform the same key function but differ in antibiotic tolerance. For example, one strain may tolerate penicillin while another tolerates tetracycline, allowing function to persist even when some members are lost. This design logic corresponds to the “complementary resistome” shown on the right side of Figure 4, where shared function is distributed across strains with different tolerance profiles. In this way, redundancy-informed design reduces dependence on any single taxon and increases the likelihood of functional persistence under antibiotic stress.

### 6.3 Clinical potential of FR scoring

In clinical treatment, the donor selection methods such as fecal microbiota transplantation have traditionally emphasized microbial diversity. However, Jiang et al. (6) pointed out that a large number of species does not necessarily mean that the transplanted microorganisms are more likely to survive. If merely the number of species is considered, some functional issues might be ignored.

Therefore, some researchers suggest including FR-related indicators in future clinical evaluations. It is useful to combine metagenomic data and structural entropy analysis methods for thoroughly assessing the donor microbiota. For instance, it is possible to analyze whether the donor samples have sufficient backup capabilities in functions such as short-chain fatty acid synthesis, bile acid metabolism, and colonization resistance. If the donor microbiota is redundant enough in these functions, the therapeutic effect of fecal microbiota transplantation may be more stable.

This approach also reflects a broader shift in microbiology, from empirical descriptions of community composition toward predictive and function-oriented frameworks for community stability. With the development of structural entropy analysis, metatranscriptomic correction, and AI-assisted functional prediction, researchers are increasingly able to evaluate microbial communities in terms of their capacity to preserve, reorganize, or recover function under antibiotic stress. In this sense, the framework proposed in this review may provide a conceptual basis for future efforts to distinguish potential redundancy from inferred active redundancy, identify the ecological contexts in which FR is most effective, and design microbiome interventions that are both functionally robust and evolutionarily sustainable. Ultimately, the value of FR lies not simply in buffering disturbance, but in revealing when, how, and at what cost bacterial communities can remain functionally stable in the face of antibiotic perturbation.
